# Optimal flickering light stimulation for entraining gamma waves in the human brain

**DOI:** 10.1038/s41598-021-95550-1

**Published:** 2021-08-10

**Authors:** Kanghee Lee, Yeseung Park, Seung Wan Suh, Sang-Su Kim, Do-Won Kim, Jaeho Lee, Jaehyeok Park, Seunghyup Yoo, Ki Woong Kim

**Affiliations:** 1 Department of Neuropsychiatry, Seoul National University, College of Medicine, Seoul National University Bundang Hospital, 82, Gumi-ro 173beon-gil, Bundang-gu, Seongnam-si, Gyeonggi-do 13620 Republic of Korea; 2 Department of Brain and Cognitive Science, Seoul National University, Seoul, Republic of Korea; 3 Department of Psychiatry, Kangdong Sacred Heart Hospital, Hallym University College of Medicine, Seoul, Republic of Korea; 4 Department of Biomedical Engineering, Chonnam National University, Yeosu, Republic of Korea; 5 School of Electrical Engineering, Korea Advanced Institute of Science and Technology (KAIST), Daejeon, Republic of Korea; 6 Department of Psychiatry, Seoul National University, College of Medicine, Seoul, Republic of Korea

**Keywords:** Cognitive ageing, Cognitive neuroscience

## Abstract

Although light flickering at 40 Hz reduced Alzheimer’s disease (AD) pathologies in mice by entraining gamma waves, it failed to reduce cerebral amyloid burden in a study on six patients with AD or mild cognitive impairment. We investigated the optimal color, intensity, and frequency of the flickering light stimulus for entraining gamma waves in young adults. We compared the event-related synchronization (ERS) values of entrained gamma waves between four different light colors (white, red, green, and blue) in the first experiment and four different luminance intensities in the second experiment. In both experiments, we compared the ERS values of entrained gamma waves between 10 different flickering frequencies from 32 to 50 Hz. We also examined the severity of six adverse effects in both experiments. We compared the propagation of gamma waves in the visual cortex to other brain regions between different luminance intensities and flickering frequencies. We found that red light entrained gamma waves most effectively, followed by white light. Lights of higher luminance intensities (700 and 400 cd/m^2^) entrained stronger gamma waves than those of lower luminance intensities (100 and 10 cd/m^2^). Lights flickering at 34–38 Hz entrained stronger and more widely spread beyond the visual cortex than those flickering at 40–50 Hz. Light of 700 cd/m^2^ resulted in more moderate-to-severe adverse effects than those of other luminance intensities. In humans, 400 cd/m^2^ white light flickering at 34–38 Hz was most optimal for gamma entrainment.

## Introduction

The power of gamma oscillations is reduced in both the animal models of Alzheimer’s disease (AD)^1,2^ and people with AD^3,4^.When beta-amyloid (Aβ) peptide was injected into the hippocampus of rodents, working memory and gamma synchronization in the medial prefrontal cortex were significantly reduced^5^. Recently, Iaccarino et al.^6^ showed that gamma waves entrained by 40 Hz flickering room light for 1 h a day for a week reduced Aβ burden by increasing amyloid endocytosis of microglia in the primary visual cortex of a transgenic AD mouse model (5XFAD). Their following study also showed that entrained gamma oscillations by a combination of 40 Hz flickering room light and 40 Hz auditory stimuli for 1 h a day for a week reduced Aβ burden in the medial prefrontal cortex and improved the recognition and spatial memory in 5XFAD mice^7^.

Gamma entrainment using flickering light stimulation (FLS) can be a promising non-invasive intervention for preventing AD or modifying the course of AD if it can reduce the Aβ burden in human brains. In a recent pilot study, 552–577 cd/m^2^ light flickering at 40 Hz for 10 consecutive days, 60 min a time, twice a day at home failed to reduce amyloid burden in the primary visual cortex, visual association cortex, lateral parietal cortex, precuneus, and posterior cingulate of six Aβ-positive patients with AD or mild cognitive impairment^8^. However, the study did not examine whether their FLS properly entrained gamma waves in the participants, leaving it for future research whether FLS failed to entrain gamma waves or gamma entrainment failed to reduce the amyloid burden in humans. The optimal parameters of FLS for entraining gamma waves in humans may differ from those in mice. In humans, the gamma wave entrained by light flickering at ≤ 54 Hz spreads to the frontocentral region while that entrained by light flickering at > 54 Hz did not^9^. The spectral power of the entrained gamma wave increased as the intensity of FLS increased^10^. Although the color (wavelength) of FLS may also influence the entrainment of gamma waves^11,12^, its effect on gamma entrainment has never been investigated. In addition, adverse effects of FLS has also never been investigated.

This study aimed to find the optimal color, intensity, and flickering frequency of FLS that can entrain gamma waves as strong and wide as possible in human brains with tolerable adverse effects.

## Results

### Entrainment of gamma oscillations

**Figure 1:**
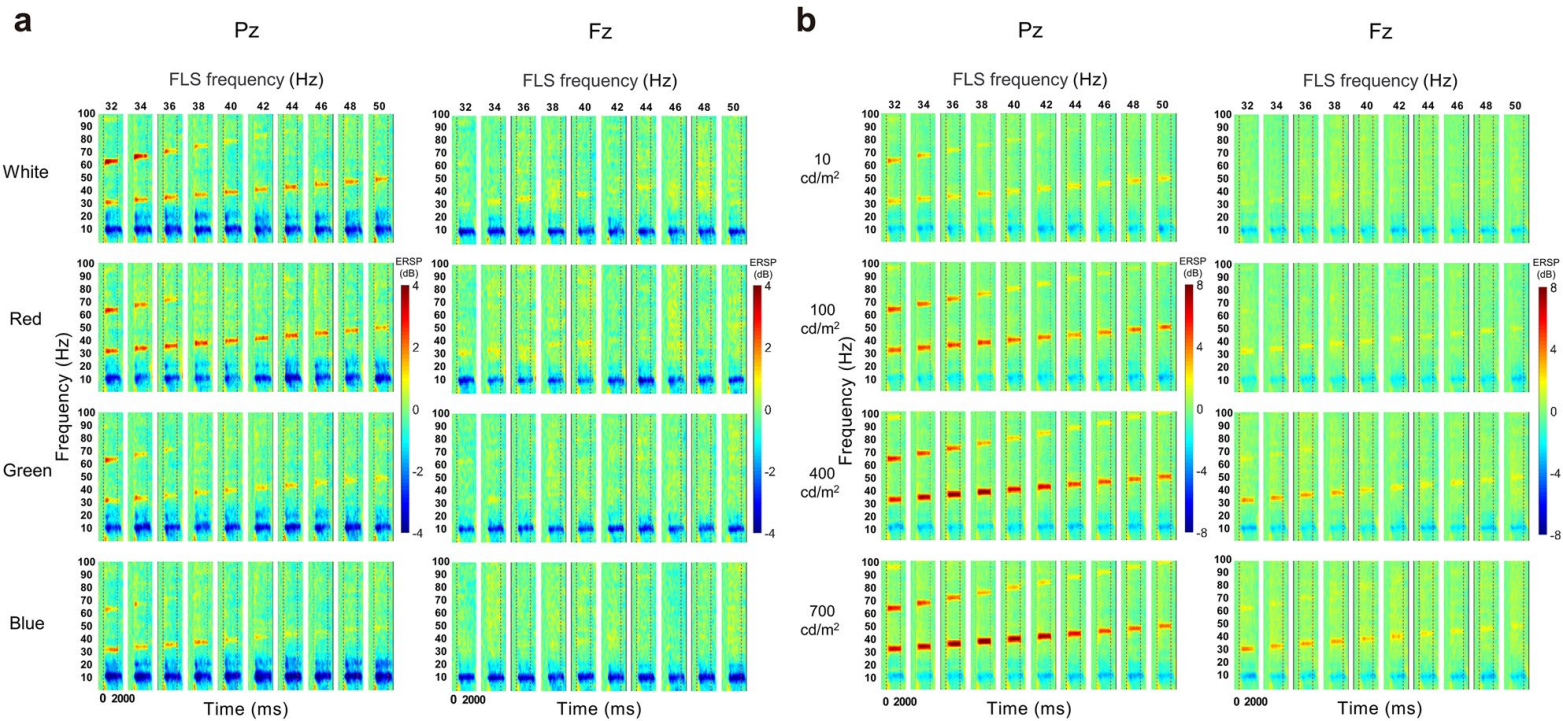
Grand-average ERSP of SSVEP induced by flickering light stimulus (FLS). Each column shows ERSP from 750 ms before the onset of FLS to 750 ms after the offset of FLS. (**A**) Comparison of grand-average ERSPs between colors. (**B**) Comparison of grand-average ERSPs between luminance intensities.

**Figure 2:**
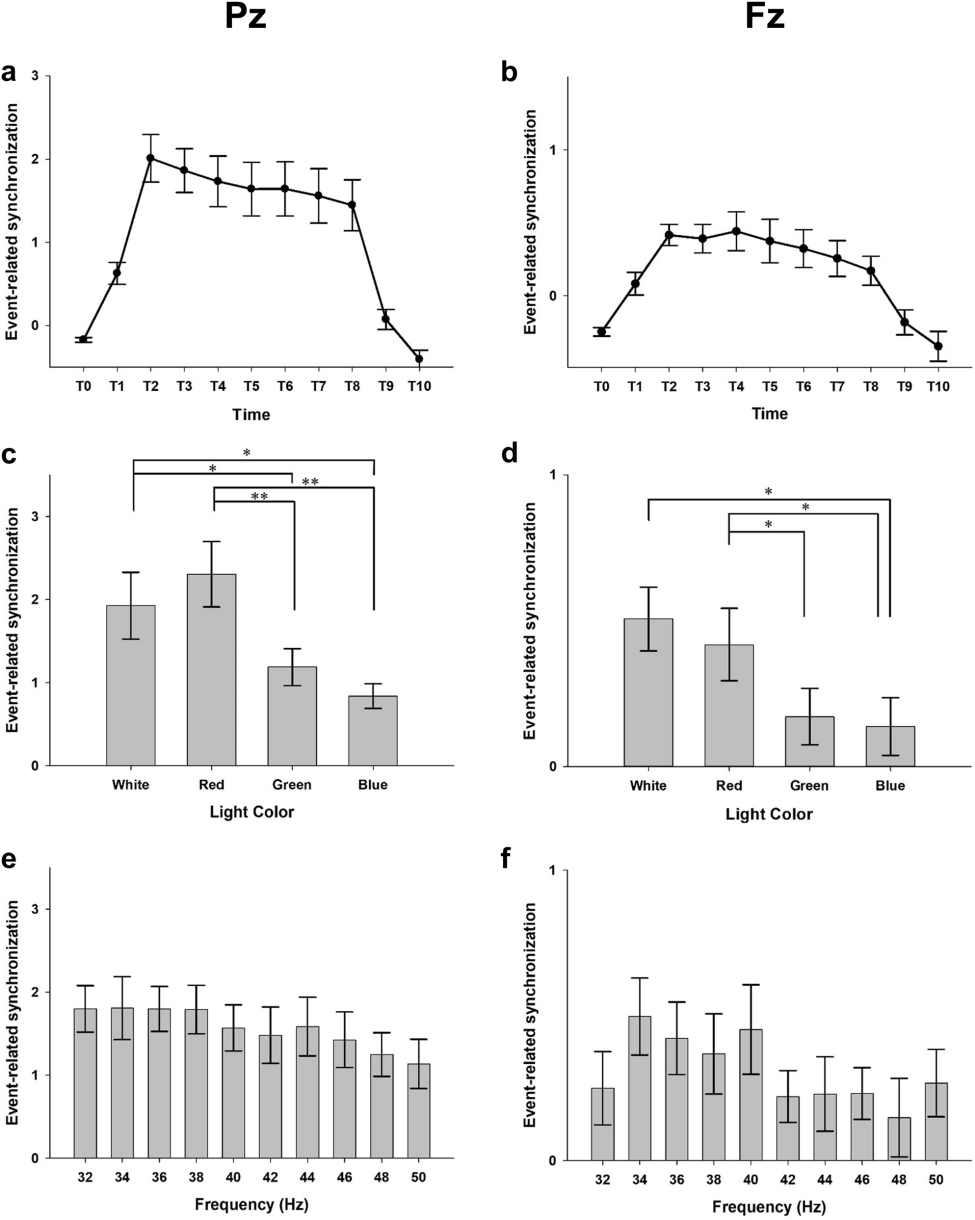
Plots of results of the repeated measures ANOVA in the EXP-1. Averaged event-related synchronization of steady-state visually evoked potentials induced by flickering light stimulus (FLS) in the first experiment. T0–T10 indicates 250 ms time windows from 250 ms before the onset of FLS to 500 ms after the offset of FLS. The main effect of the time window on ERS at Pz (**A**) and Fz (**B**). The main effects of the color of FLS on ERS at Pz (**C**) and Fz (**D**). The main effect of flickering frequency of FLS on ERS at Pz (**E**) and Fz (**F**). Error bars indicate standard errors. *p < 0.05, **p < 0.01, ***p < 0.001, by repeated measures analysis of variance.

**Figure 3:**
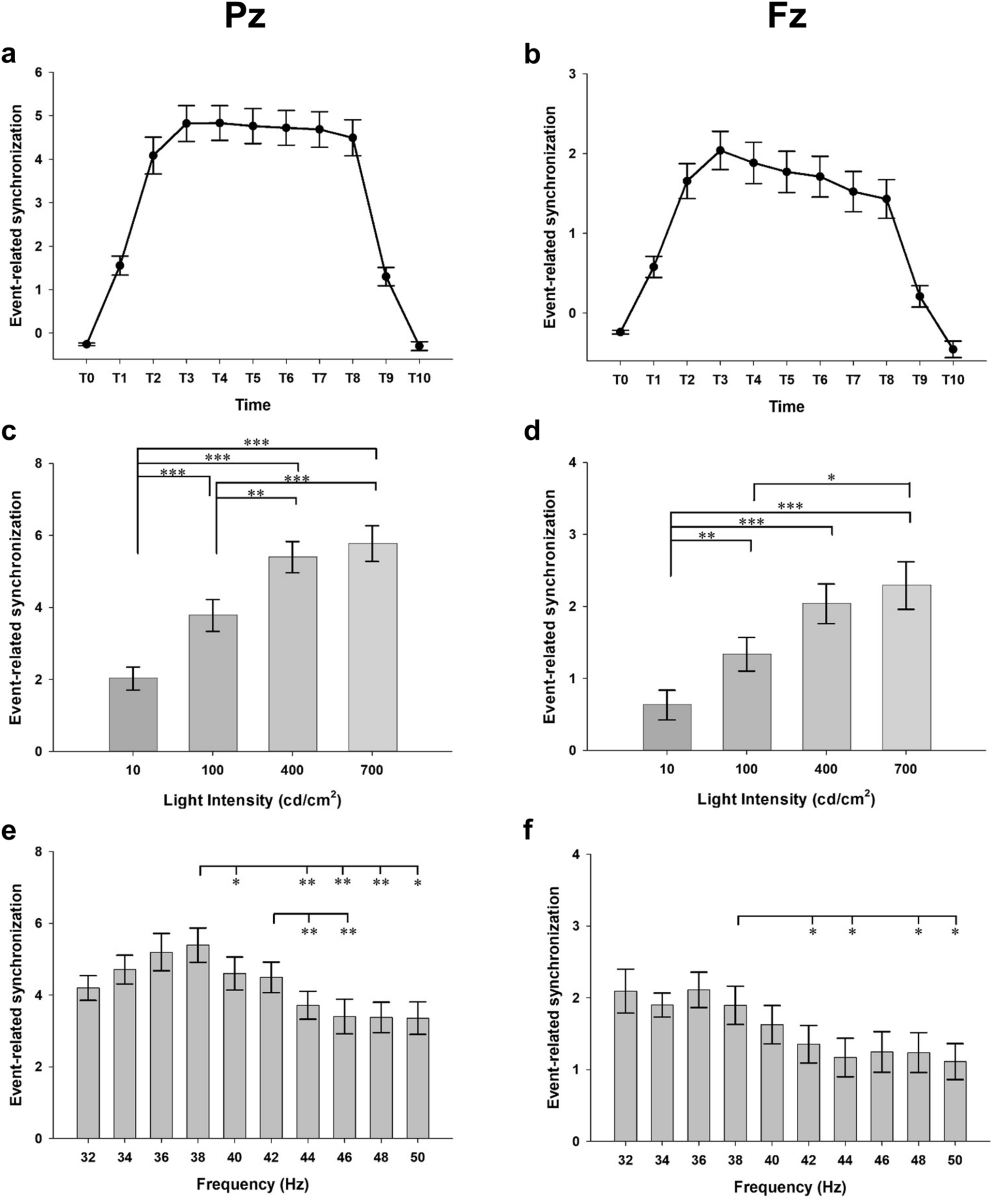
Plots of results of the repeated measures ANOVA in the EXP-2. Averaged event-related synchronization of steady-state visually evoked potentials induced by flickering light stimulus (FLS) in the second experiment. T0–T10 indicates 250 ms time windows from 250 ms before the onset of FLS to 500 ms after the offset of FLS. The main effect of the time window on ERS at Pz (**A**) and Fz (**B**). The main effects of the luminance intensity of FLS on ERS at Pz (**C**) and Fz (**D**). The main effect of flickering frequency of FLS on ERS at Pz (**E**) and Fz (**F**). Error bars indicate standard errors. *p < 0.05, **p < 0.01, ***p < 0.001, by repeated measures analysis of variance.

In both the experiments, the increase in the spectral power of SSVEP at the fundamental and harmonic frequencies of FLS started after FLS onset, lasted during the FLS, and diminished after FLS offset (Fig. 1). The main effect of the time window on ERS was significant in both EXP-1 (F_10, 150_ = 31.145, *p* < 0.001 at Pz, Fig. 2A; F_10, 150_ = 12.731, *p* < 0.001 at Fz, Fig. 2B) and EXP-2 (F_10, 150_ = 134.172, *p* < 0.001 at Pz, Fig. 3A; F_10, 150_ = 50.869, *p* < 0.001 at Fz, Fig. 3B).

In response to FLS, high gamma band brainwaves of 64 Hz ~ 100 Hz, which are harmonic frequencies of 32 Hz ~ 50 Hz, also increased in response to the light stimulation of 32 Hz ~ 50 Hz at both Pz and Fz (*p* < 0.001) while alpha band brainwaves of 9 Hz ~ 13 Hz decreased both Pz and Fz (*p* < 0.001). There were no changes in theta band and beta band brainwaves during FLS (*p* > 0.1).

### Effect of light color

In the EXP-1 (Fig. 2C, D), the main effects of the color of FLS on ERS were significant (F_3, 45_ = 12.599, *p* < 0.001 at Pz; F_3, 45_ = 6.736, *p* < 0.01 at Fz). ERS induced by red FLS and white FLS was higher than that induced by green or blue FLS (*p* < 0.05 for white FLS; *p* < 0.01 for red FLS at Pz; *p* < 0.05 for both white and red FLS at Fz). However, the differences in the ERS between red and white FLSs were not statistically significant at both Pz and Fz (*p* = 0.117 at Pz; *p* = 1.000 at Fz).

### Effect of light intensity

In the EXP-2 (Fig. 3C, D), the main effects of the luminance intensity of FLS on ERS were significant (F_3, 45_ = 49.156, *p* < 0.001 at Pz, Fig. 3C; F_3, 45_ = 17.742, *p* < 0.001 at Fz, Fig. 3D). The ERS induced by 700 cd/m^2^ and 400 cd/m^2^ FLSs was higher than that induced by 100 cd/m^2^ and 10 cd/m^2^ FLSs (*p* < 0.01 for 400 cd/m^2^ FLS, *p* < 0.001 in 700 cd/m^2^ FLS at Pz; *p* < 0.001 in 400 cd/m^2^ FLS, *p* < 0.05 in 700 cd/m^2^ FLS at Fz). However, the differences in the ERS between 700 cd/m^2^ and 400 cd/m^2^ FLSs were not statistically significant (*p* = 0.970 at Pz; *p* = 1.000 at Fz).

### Effect of flickering frequency of light

In both experiments, the fundamental and harmonic responses became weaker with the increase in the flickering frequency over 40 Hz regardless of the color and intensity of FLS. In the EXP-1, the main effect of flickering frequency (F_9, 135_ = 1.998, *p* = 0.131 at Pz; F_9, 135_ = 1.606, *p* = 0.154 at Fz) and its interaction with the light color on ERS (F_27, 405_ = 1.261 *p* = 0.269 at Pz; F_27, 405_ = 1.061, *p* = 0.396 at Fz) were not statistically significant (Fig. 2E, F). In the EXP-2, however, the main effect of the flickering frequency (F_9, 135_ = 9.042, *p* < 0.001 at Pz; F_9, 135_ = 6.492 *p* < 0.001 at Fz) was significant. ERS was highest at 38 Hz, followed by 36 Hz and 34 Hz at Pz and was highest at 36 Hz, followed by 38 Hz and 34 Hz at Fz (Fig. 3E, F). Moreover, the interaction between the flickering frequency and luminance intensity was also significant (F_27, 405_ = 5.514, *p* < 0.001 at Pz; F_27, 405_ = 1.794, *p* = 0.78 at Fz). ERS was highest at 36 Hz, followed by 38 Hz and 34 Hz for 400 cd/m^2^ FLS and was highest at 38 Hz, followed by 36 Hz and 40 Hz for 700 cd/m^2^ FLS.

### The propagation of entrained gamma waves

**Figure 4:**
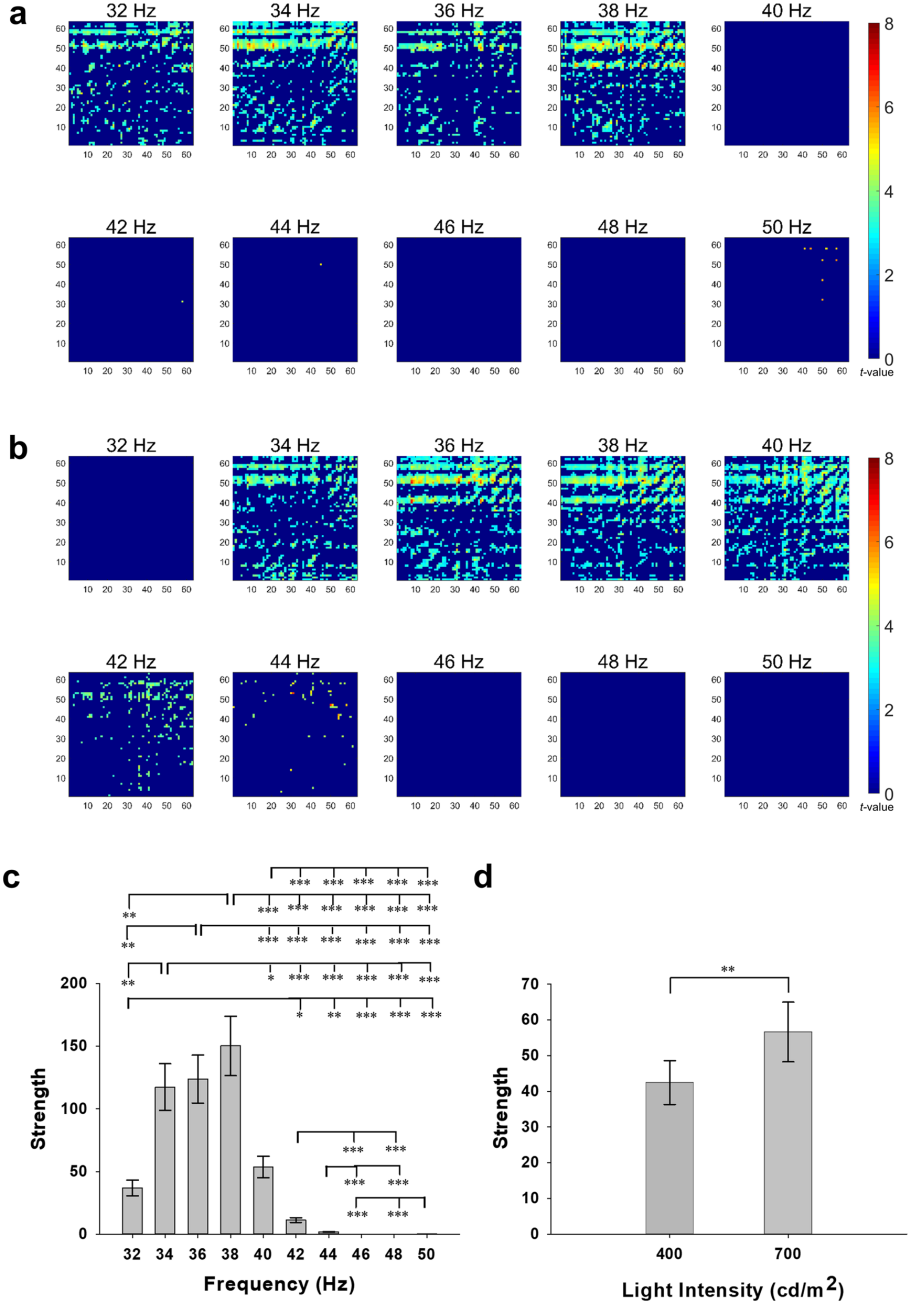
The spectral Granger causality of entrained gamma EEG. Changes in the gamma wave spectral Granger causality of 1953 connections between 63 electroencephalogram (EEG) electrodes induced by flickering light stimulus in the second experiment (**A**, **B**) and the averaged strength of the occipitoparietal to frontotemporal gamma wave connections estimated using spectral Granger causality analysis (**C**, **D**). (**A**) 400 cd/m^2^ and (**B**) 700 cd/m^2^. The left-upper side of each matrix represents the occipitoparietal to frontotemporal connections. The numbers of EEG electrodes from 1 to 63 correspond to Fp1, Fp2, AF7, AF3, AFz, AF4, AF8, F7, F5, F3, F1, Fz, F2, F4, F6, F8, FT9, FT7, FC5, FC3, FC1, FC2, FC4, FC6, FT8, FT10, T7, C5, C3, C1, Cz, C2, C4, C6, T8, TP9, TP7, CP5, CP3, CP1, CPz, CP2, CP4, CP6, TP8, TP10, P7, P5, P3, P1, P2, P4, P6, P8, PO7, PO3, POz, PO4, PO8, O1, Oz, and O2, respectively. Error bars indicate standard errors. *p < 0.05, **p < 0.01, ***p < 0.001.

In the sGC analysis, the number of edges with significantly increased parietooccipital to frontotemporal connectivity of entrained gamma waves with the same frequency of administered FLS was higher than that in the rsEEG when the flickering frequency of FLS ranged from 32 to 38 Hz for 400 cd/m^2^ FLS and from 34 to 40 Hz for 700 cd/m^2^ FLS (Fig. 4A, B).

The main effects on connectivity strength of the flickering frequency (F_9, 135_ = 34.982, *p* < 0.001, Fig. 4C) and the intensity (F_1, 15_ = 10.306, *p* < 0.001, Fig. 4D), and their interaction (F_9, 135_ = 20.591, *p* < 0.001) were all significant. The strength of connectivity induced by 700 cd/m^2^ FLS was higher than that induced by 400 cd/m^2^ FLS. The strength of connectivity induced by 34–38 Hz FLS was significantly higher than that induced by FLS of other flickering frequencies. The strength of connectivity was highest at 34 Hz, followed by 38 Hz and 36 Hz at 400 cd/m^2^ FLS and highest at 38 Hz, followed by 36 Hz and 40 Hz at 700 cd/m^2^ FLS.

### Adverse effects

The frequencies of dazzling and asthenopia were different between the colors of FLS in the EXP-1 (F_3,45_ = 3.12, *p* < 0.05 for dazzling; F_3,45_ = 5.43, *p* < 0.05 for asthenopia, Table 1). Asthenopia was more common with red and white FLSs when compared with green FLS (*p* < 0.05). However, the severity of all adverse effects was mild (< 3). In the EXP-2, the frequencies of fatigue, dazzling, asthenopia, and ocular pain were different between the luminance intensities of FLS (F_3,45_ = 7.85, *p* < 0.005 for fatigue; F_3,45_ = 47.00, *p* < 0.001 for dazzling; F_3,45_ = 15.66, *p* < 0.001 for asthenopia; F_3,45_ = 9.23, *p* < 0.005 for ocular pain). Fatigue, dazzling, asthenopia, and ocular pain were more common with 700 cd/m^2^ FLS than with FLS of other intensities. The severities of dazzling and asthenopia were moderate to severe (> 3) at 700 cd/m^2^ FLS. Dazzling and asthenopia were more common with 400 cd/m^2^ than with 10 cd/m^2^ FLS.

**Table 1:** Self-reported adverse effects of flickering light stimulation.

	Color						Intensity					
	White	Red	Green	Blue	F	Post hoc	10 cd/m^2^	100 cd/m^2^	400 cd/m^2^	700 cd/m^2^	F	Post hoc
Fatigue	2.1 (1.8)	2.1 (1.8)	1.3 (1.9)	1.7 (1.9)	1.84	–	1.2 (1.3)	2.0 (1.7)	2.1 (1.8)	2.8 (2.0)	7.85**	h > e, g
Headache	0.1 (0.3)	0.1 (0.3)	0.2 (0.5)	0 (0)	1.00	–	0 (0)	0.1 (0.3)	0.3 (0.9)	0.6 (1.1)	3.02	–
Dizziness	2.3 (2.0)	1.3 (1.4)	1.6 (1.5)	1.4 (1.6)	2.75	–	1.4 (1.3)	1.6 (1.1)	1.2 (1.2)	1.2 (1.2)	1.37	–
Dazzling	1.9 (1.7)	2.6 (1.8)	1.4 (1.2)	2.3 (2.1)	3.12*	–	1.4 (1.0)	2.7 (1.4)	3.6 (1.5)	4.7 (1.3)	47.00***	h > g > f > e
Asthenopia	2.3 (1.3)	2.6 (1.6)	1.4 (1.2)	2.4 (1.9)	5.43*	a, b > c	1.6 (1.1)	2.2 (1.6)	2.7 (1.7)	3.6 (1.9)	15.66***	h, g > e, h > f
Ocular pain	0.4 (0.7)	0.6 (1.25)	0.1 (0.3)	0.6 (1.2)	1.78	–	0.3 (0.8)	0.6 (1.0)	1.0 (1.4)	1.7 (1.8)	9.23**	h > e, f, g

a, white; b, red; c, green; d, blue; e, 10 cd/m^2^; f, 100 cd/m^2^; g, 400 cd/m^2^; h, 700 cd/m^2^.

*p < 0.05, **p < 0.01, ***p < 0.001 by repeated measures analysis of variance.

## Discussion

Our study demonstrated that gamma entrainment was considerably influenced by the color, luminance intensity, and flickering frequency of FLS in humans. Lights with long wavelengths (red or white) entrained stronger gamma oscillation than those with short wavelengths (green or blue). White light with high luminance intensities (700 cd/m^2^ or 400 cd/m^2^) entrained stronger gamma oscillation than that with low intensities (100 cd/m^2^ or 10 cd/m^2^). White lights flickering at 34–38 Hz entrained stronger and more widely spread gamma oscillations than those flickering at other frequencies. Adverse effects were more common and moderate to severe in the FLS of 700 cd/m^2^ than those with lower intensities.

FLS with long wavelengths induced stronger SSVEP than FLS with shorter wavelengths in humans^13,14^. In humans, retinal cones are responsible for color vision, and long-wavelength sensitive cones are denser than medium-wavelength and short-wavelength sensitive cones. The ratio of red, green, and blue cones in the retina is 11:5:1^15,16^. Most of the cones, which are primarily located in the fovea of ​​the retina, are connected to only one bipolar cell, which in turn connects to only one midget ganglion cell^17,18^. Lights with longer wavelengths can reach a wider primary visual cortex area and may entrain gamma waves stronger than those with shorter wavelengths. In the current study, ERS entrained by white FLS was slightly lower than that entrained by red FLS at Pz but was comparable to that entrained by red FLS at Fz. However, Bieger and colleagues^19^ reported that white FLS using white-black contrast showed a faster information transfer rate of SSVEP-based brain-computer interface than FLSs using red, green, or blue colors. White FLS may entrain gamma waves as strong as or even better than red FLS if its contrast is optimized.

FLS with high luminance intensity and/or contrast was also found to induce stronger SSVEP than FLS with low luminance intensity and/or contrast in humans^20–22^. The amplitudes of SSVEP entrained by 100 cd/m^2^, 400 cd/m^2^, and 700 cd/m^2^ FLS were 94.1%, 180.4%, and 201.9% higher than those entrained by 10 cd/m^2^ FLS in our study, respectively. In previous studies, 1,000 cd/m^2^ FLS entrained 13% stronger SSVEP than 400 cd/m^2^ FLS^23^, and 1400 cd/m^2^ FLS entrained gamma waves more strongly and widely than 700 cd/m^2^ FLS^10^.

Tsoneva et al.^9^ reported that 40–50 Hz FLS entrained stronger SSVEP than 52–58 Hz FLS, and 40–54 Hz FLS entrained gamma waves more widely than 54–58 Hz FLS in humans. These results suggested that lights flickering at a lower frequency of gamma range maybe better for gamma entrainment than those at a higher frequency; thus, it raised a further question of whether lights flickering at < 40 Hz may be even better for gamma entrainment than those with flickering at 40–50 Hz. In our study, we found that FLS of 34–38 Hz could entrain gamma waves more strongly and widely than FLS of 40–48 Hz in humans. Although the studies on AD mice employed 40 Hz FLS^6,7,24^, future clinical studies on humans need to consider employing FLS of 34–38 Hz rather than 40 Hz because lights flickering at 30 Hz also prevented the neurodegeneration of pyramidal neurons in hippocampal CA of ischemic mouse models^25^.

In AD mice, visual stimulation alone could induce gamma waves and reduce AD pathologies in brain regions beyond the visual cortex^24^, and auditory stimulation alone could induce gamma waves and reduce AD pathologies in the brain regions beyond the auditory cortex^7^. These results indicate that the gamma waves entrained in the sensory cortex by sensory stimuli can propagate to other brain regions and reduce AD pathologies. Tsoneva et al.^9^ demonstrated that gamma waves entrained by lights flickering at < 54 Hz spread from the posterior to the anterior regions of the human brain using the topographical changes of SSVEP. However, the topographical changes of SSVEP do not necessarily indicate the propagation of gamma waves from visual cortex to other brain regions because gamma waves can also be directly induced in other brain regions during FLS. For example, attentional gamma response was induced in the frontal eye field during visual stimulation in macaque monkeys^26^. Therefore, we examined the propagation of gamma waves using sGC analysis. Since sGC describes whether the preceded EEG signal in channel X can predict the following EEG signal in channel Y^27,28^, we can separate the gamma waves from the visual cortex from those to the visual cortex^29,30^. The numbers of nodes where the gamma waves in the occipital region were entrained by FLSs of 34 Hz, 36 Hz, and 38 Hz were 1.7, 1.6, and 2.1 times higher and their strengths of connections were 2.2, 2.3, and 2.8 times higher than those entrained by FLS of 40 Hz, respectively. This was the case for both 400 cd/m^2^ and 700 cd/m^2^ FLSs. These results indicate that the visual SSVEP entrained by FLS of 34–38 Hz may spread more strongly and widely than that by FLS of 40 Hz when the luminance intensity is ≥ 400 cd/m^2^. Since the gamma waves entrained by FLS of 34–38 Hz successfully propagates to the frontal and temporal areas, we may try to reduce AD pathologies using visual stimulus only without any additional sensory stimulus if its parameters are optimized. The 40 Hz FLS, which was employed in a previous preliminary clinical study on six MCI and AD patients^8^, might have failed to make the entrained gamma waves propagate to the frontal and temporal areas.

In our study, 700 cd/m^2^ FLS showed more adverse effects than 400 cd/m^2^ FLS, and some of their adverse effects were moderate to severe. Although 400 cd/m^2^ entrained gamma waves slightly less than FLS 700 cd/m^2^ FLS, it also entrained strong gamma waves beyond visual cortex and was more tolerable than FLS 700 cd/m^2^ FLS. To ensure that gamma waves are properly entrained in target brain regions and that people can use enough time with tolerable adverse effects, it is important to control the light intensity accurately. In this sense, a spectacle-type FLS equipment as we employed in the current study is likely to be more effective than a light-type equipment as a therapeutic medical device. In conclusion, 400 cd/m^2^ white light flickering at 34–38 Hz seems to be the most optimal for entraining strong and widespread gamma waves with tolerable adverse effects in humans.

## Methods

### Participants

We enrolled 19 young healthy volunteers (11 men and eight women, age: 24.1 ± 3.6 years). All participants had a normal or corrected-to-normal vision. None of them reported a previous history of major psychiatric or neurological disorders, including epilepsy. Sixteen participants (nine men and seven women, age: 24.0 ± 3.7 years) were included in the final analysis after excluding three participants: one dropped out during the experiments due to severe glare and other two had excessive noise in their electroencephalogram (EEG) data. All participants provided a written informed consent. All procedures were performed in accordance with the Declaration of Helsinki and approved by the institutional review board of Seoul National University Bundang Hospital (IRB No.: B-1904-534-303).

### FLS

FLS was delivered using a pair of organic light-emitting diodes (OLED) panels (4.7 cm × 4.7 cm; color temperature 3000 K; LG Display Co., Ltd., Seoul, Korea) attached to an eyeglass. Voltage-luminance characteristics of OLED panels were calibrated using calibrated spectroradiometer (CS2000, Konica-Minolta Inc. Tokyo, Japan) at voltage-controlled mode using precise source measurement unit (Keithley 2400, Tektronix Inc., Beaverton, OR, USA).

The OLED panels were driven with a square wave using a function generator (TG 5012A, Aim & Thurlby Thandar Instruments, Huntingdon, Cambridgeshire, UK) with 100% modulation depth and 50% duty cycle. The error of voltage fluctuation was controlled under $\pm$ 5 mV and the error of luminance is estimated less than 1 cd/m^2^ for 10 cd/m^2^ light intensity of four colors and less than 5% for 10, 100, 400, 700 cd/m^2^ light intensity of white OLED (Fig. S1, S2). The amplitude and frequency of the square wave were modulated to change the luminance and frequency of FLS using an in-house LabVIEW program (National Instruments Corporation, Austin, TX, USA). The colors of FLS were controlled using optical color filters (KODAK WRATTEN 2, Eastman Kodak Company; No. 25 for red, No. 58 for green, and No. 47 for blue). Red, green, blue, and white colors had their peak wavelengths at 700 nm, 530 nm, 440 nm, and 610 nm, respectively. The intensities of FLS were controlled by changing the supply voltage of the OLED for each color. For 10 cd/m^2^, 7.19 V, 7.25 V, 7.99 V, and 7.04 V were supplied for red, green, blue, and white FLSs, respectively. For 100 cd/m^2^, 400 cd/m^2^, and 700 cd m^2^ of white FLS, 7.38 V, 7.71 V, and 7.91 V were supplied, respectively. For all colors and intensities, FLS was provided at 10 distinctive flickering frequencies (32 Hz, 34 Hz, 36 Hz, 38 Hz, 40 Hz, 42 Hz, 44 Hz, 46 Hz, 48 Hz, and 50 Hz) in the gamma-band range. The distance between OLED panel and the cornea was set to 2 cm. The subtended angle seen by the center point of cornea for the middle points of edges and the vertex point of OLED panel were 1.73 and 2.06 radians, respectively.

### Experimental design

**Figure 5:**
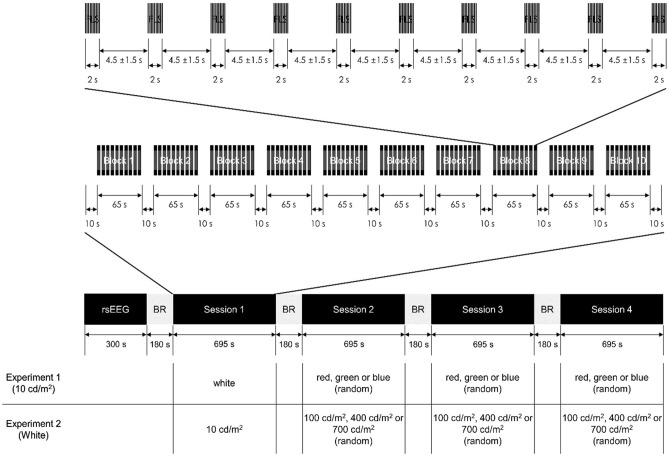
Experimental procedures. *FLS* flickering light stimulus; *rsEEG* resting-state electroencephalogram; *BR* break.

The study consisted of two experiments (Fig. 5): the first experiment (EXP-1) for identifying the optimal color of FLS for entraining gamma waves and the second experiment (EXP-2) for identifying the optimal intensity of FLS for entraining gamma waves. From both experiments, we identified the optimal frequency of FLS for entraining gamma waves.

Each experiment was conducted twice for each participant, and the inter-experiment intervals were 7–10 days. Participants were instructed not to drink alcohol 24 h before all experiments and fast for at least 2 h before all experiments.

Each experiment consisted of a 5-min resting phase for recording resting-state EEG (rsEEG) and four experimental sessions for recording steady-state visually evoked potentials (SSVEP). Each session consisted of ten blocks, and each block consisted of ten FLS trials of a given color, intensity, and flickering frequency. A 10-s break was placed before and after each block. Each FLS was presented for two seconds, and the inter-FLS interval was randomly given from 3 to 6 s (4.5 ± 1.5 s). The flickering frequencies of FLS in each block were one of the ten frequencies from 32 to 50 Hz with a 2-Hz interval, and the order of flickering frequency of the 10 blocks was random. In the EXP-1, the color of 10 cd/m^2^ FLS in the first session was always white, and those of the following sessions were randomly assigned among other colors. In the EXP-2, the intensity of white FLS in the first session was always 10 cd/m^2^, and those of the following sessions were randomly assigned among other intensities.

In each experiment, rsEEG was recorded for 5 min during eye-closed. In each session, EEG was recorded for 11 min 55 s from 10 s before the first block. We instructed participants to keep their eyes open while FLS was performed. After each session, participants were asked to rate the severity of six adverse effects of FLS (fatigue, headache, dizziness, dazzling, asthenopia, and ocular pain) using a 7-point Likert-type scale from 0 (not at all) to 6 (extremely severe) by themselves.

### EEG recording and preprocessing

We recorded EEG using 64 Ag–AgCl electrodes attached to an elastic cap (Easycap, EASYCAP GmbH, Munich, Germany) according to the extended International 10–20 System. The reference electrode was FCz. We placed the ground electrode on the forehead, and attached a pair of electrooculogram electrodes below and above the left eye. The impedance of electrodes was 10 kΩ or less during the recording. We recorded EEG signal with 1000 Hz sampling rate using a 24-bit actiCHamp DC amplifier and BrainVision Recorder (Brain Products Gmbh, Gliching, Germany). The stimulus markers were delivered from the FLS control system and were synchronized with the recording.

All preprocessing and analysis procedures were conducted using EEGLAB^31^, and BSMART^32^ toolbox. We filtered the recorded signals with a 1 Hz high-pass finite impulse response filter and a 60 Hz notch filter and then applied a common average reference. An independent component analysis was performed to remove ocular artifacts from the EEG signal. We segmented 5-min rsEEG recordings into 1500-ms epochs, and randomly selected 10 artifact-free epochs from them at each visit. We obtained a 4000-ms epoch from − 1000 to 3000 ms of each FLS onset.

### Analyses

To find the optimal color, intensity, and frequency of FLS for entraining gamma waves, the spectral power change of EEG induced by FLS was analyzed using event-related spectral perturbation (ERSP) in each block. We calculated the event-related desynchronization/event-related synchronization (ERD/ERS) value by averaging them from 10 FLS trials in each block to get a normalized averaged spectral power change induced by a given color, intensity, and frequency of FLS.

The time-associated change of power spectrum was also calculated by averaging ERS of 11 successive, non-overlapping, 250-ms subwindows from 250 ms before FLS onset to 2500 ms after FLS onset in each block (T0–T10). To examine whether FLS entrains the gamma wave, we compared ERS of 11 blocks. To examine the effect of frequency, color, or intensity, we used averaged ERS during T1–T8.

To examine whether gamma waves entrained in the occipital cortex propagate to other brain areas, the spectral Granger causality (sGC) of gamma waves was compared in each block of the white FLS of 400 cd/m^2^ and 700 cd/m^2^ in the EXP-2 to that of gamma waves of the same frequency in rsEEG^33–35^. We compared the sGC of rsEEG with that of SSVEP during FLS using paired *t*-test with a false discovery rate corrected *p* value of 0.05. We constructed an adjacency matrix of a given intensity and flickering frequency of FLS using the edges that were found to be significantly different between rsEEG and SSVEP. We employed graph theory measures to compare the network structures quantitatively between different conditions of light intensity and frequency^36,37^.

Repeated-measures ANOVAs (alpha = 0.05) for ERS, connectivity strength, and adverse effects were computed using SPSS version 22 (IBM Corporation, Armonk, NY). Contrast comparisons were used for *post-hoc* tests.

## Supplementary Information

Supplementary Information.

## Abbreviations

ERD/ERS: Event-related desynchronization/event-related synchronization
ERSP: Event-related spectral perturbation
FLS: Flickering light stimulation
OLED: Organic light-emitting diodes
rsEEG: Resting-state electroencephalogram
SSVEP: Steady-state visually evoked potentials
sGC: Spectral granger causality
