# Supplementary material for: Optimal flickering light stimulation for entraining gamma waves in the human brain
Source: Sci Rep. 2021 Aug 10;11:16206. doi: 10.1038/s41598-021-95550-1 (PMC8355349; doi:10.1038/s41598-021-95550-1)
Supplement: Supplementary file 1 — Supplementary Information. [file 41598_2021_95550_MOESM1_ESM.pdf]

Title: Optimal flickering light stimulation for entraining gamma waves in the human brain.

Kanghee Lee[1,†], Yeseung Park[1,2,†], Seung Wan Suh[1], Sang-Su Kim[3], Do-Won Kim[3], Jaeho Lee[4], Jaehyeok Park[4], Seunghyup Yoo[4], Ki Woong Kim[1,2,5,*]

[1]Department of Neuropsychiatry, Seoul National University Bundang Hospital, Seongnam, Republic of Korea

[2]Department of Brain and Cognitive Science, Seoul National University, Seoul, Republic of Korea

[3]Department of Biomedical Engineering, Chonnam National University, Yeosu, Republic of Korea

[4]School of Electrical Engineering, Korea Advanced Institute of Science and Technology (KAIST), Daejeon, Republic of Korea

[5]Department of Psychiatry, Seoul National University, College of Medicine, Seoul, Republic of Korea

[†]These authors have contributed equally to this work and share first authorship

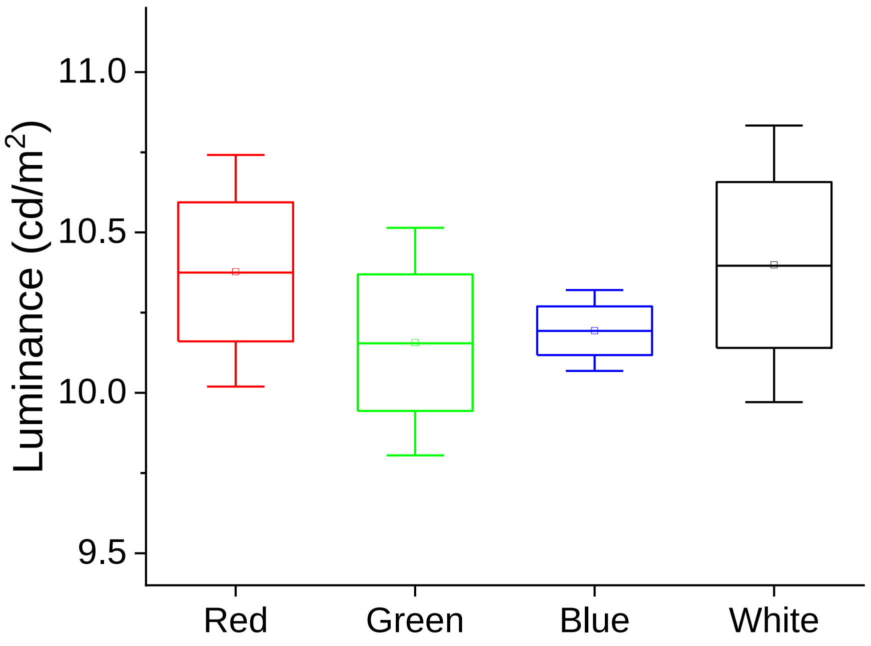

Supplementary Figure 1. Estimated luminance and error bound at 10cd/m^2. Dots, boxes, error bars indicate median, 25~75% probability distribution of luminance, 1.5 interquartile range, respectively.

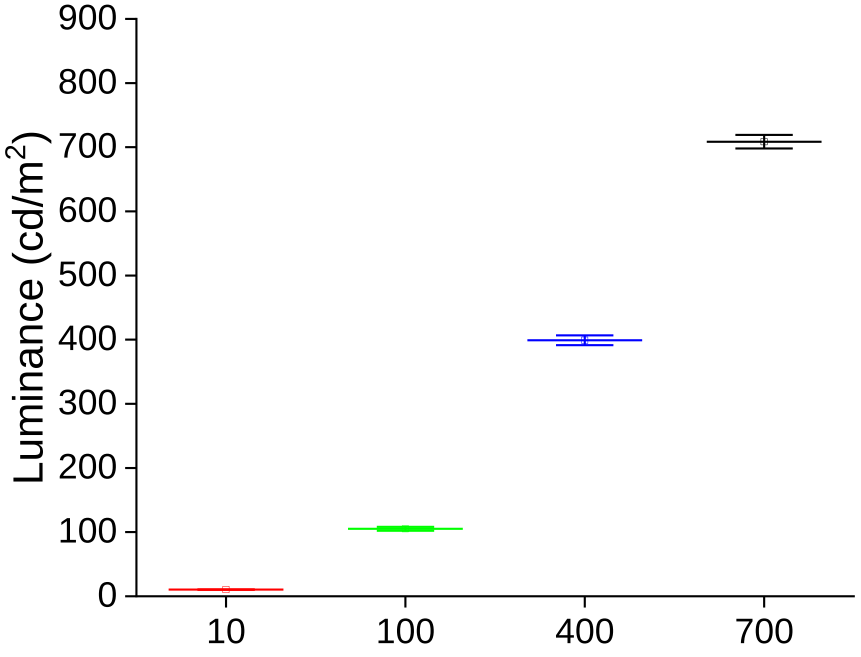

Supplementary Figure 2. Estimated luminance and error bound of white OLED at various light intensity. Dots and error bars indicate median and 1.5 interquartile range, respectively.

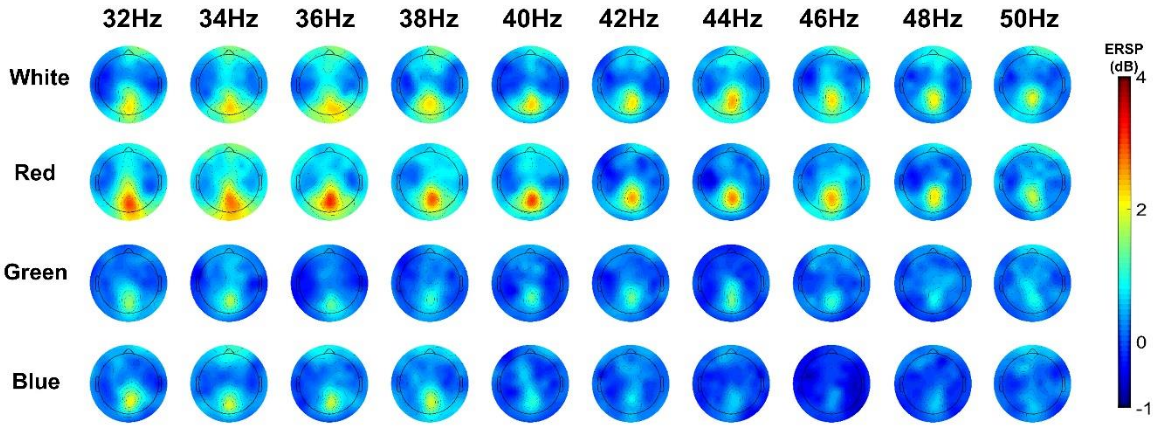

Supplementary Figure 3. Topography of the gamma wave entrained by flickering light stimulation of different colors in the experiment 1

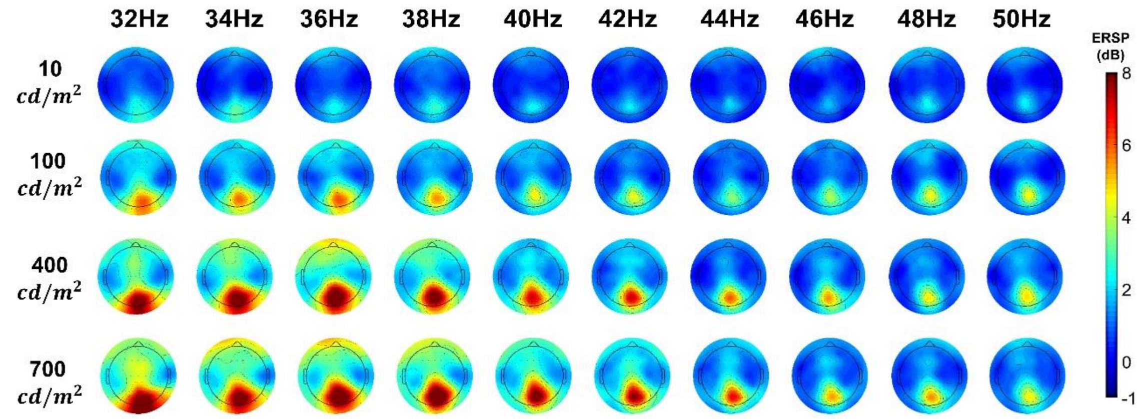

Supplementary Figure 4. Topography of the gamma wave entrained by flickering light stimulation of different luminance intensities in the experiment 2
